# Acute Toxic and Genotoxic Effects of Aluminum and Manganese Using In Vitro Models

**DOI:** 10.3390/toxics9070153

**Published:** 2021-06-30

**Authors:** Luiza Flavia Veiga Francisco, Debora da Silva Baldivia, Bruno do Amaral Crispim, Syla Maria Farias Ferraz Klafke, Pamella Fukuda de Castilho, Lucilene Finoto Viana, Edson Lucas dos Santos, Kelly Mari Pires de Oliveira, Alexeia Barufatti

**Affiliations:** 1 Faculty of Exact Sciences and Technology, Federal University of Grande Dourados, Dourados 79.804-970, Mato Grosso do Sul, Brazil; luizaveiga10@hotmail.com; 2 Faculty of Biological and Environmental Sciences, Federal University of Grande Dourados, Dourados 79.804-970, Mato Grosso do Sul, Brazil; deborabaldivia@outlook.com.br (D.d.S.B.); brunocrispim.bio@gmail.com (B.d.A.C.); sylamklafke@gmail.com (S.M.F.F.K.); lucilenefinoto@hotmail.com (L.F.V.); edsonsantos@ufgd.edu.br (E.L.d.S.); kellyoliveira@ufgd.edu.br (K.M.P.d.O.); 3 Postgraduate Program in Health Science, Federal University of Grande Dourados, Dourados 79.804-970, Mato Grosso do Sul, Brazil; pamellafcastilho@gmail.com

**Keywords:** metals, cytotoxicity, micronucleus, comet assay, Ames test, CHO cells

## Abstract

The objective of this study was to use the same concentrations of aluminum (Al) and manganese (Mn) detected previously in groundwater above those permitted by Brazilian law and assess their cytotoxic and genotoxic effects in hamster ovary cell lines and their mutagenic effects through the *Salmonella* microsome assay. Chinese hamster ovary (CHO) and CHO-XRS5 cells were treated with different concentrations of Al and Mn (0.2 to 2.0 mg/L and 0.1 to 3.0 mg/L, respectively). The Ames test was used to analyze the concentrations of Al and Mn ranging from 0.025 to 1.0 mg/L and 0.0125 to 1.5 mg/L, respectively. Both metals showed cytotoxic effects on both cell lines and two bacterial strains (TA98 and TA100). The genotoxic effects of the highest concentrations of Al and Mn in cell lines showed nuclear buds, micronuclei, and DNA damage; however, none of the concentrations showed a positive mutagenic response in the Ames test. This is one of the few studies to demonstrate the cytotoxic effects of Al and Mn through the Ames test. In addition, the metals caused genomic instability in cell lines. Therefore, this study may help hasten the review of established regulatory standards for human consumption of groundwater.

## 1. Introduction

Exposure to metals in the environment at concentrations above of the values considered safe by legislation can adversely affect organisms [1,2,3]. Metal contamination in the environment occurs from both natural sources, such as rocks and volcanoes, and anthropogenic sources, such as through industrial processes, mining, pesticides, medication, and water treatment [4,5]. Contaminated food and drinking water are the most common sources of human exposure to metals [6,7]. Food contamination is mainly caused by the use of pesticides containing metals in agriculture, whereas contamination of water is mainly caused by inadequate disposal of these metals in the soil due to agricultural, industrial, and landfill activities, which can contaminate surface and groundwater through percolation and leaching processes [8].

Aluminum (Al) is the most abundant metal on the Earth’s surface. It is used on a large scale for food packaging and in kitchen utensils [9], which can act as sources of Al toxicity to humans as Al does not have a biological role in living organisms [10]. Manganese (Mn) is the fifth most abundant metal and is considered an essential element for humans because it plays an important role in several physiological processes, such as the regulation of reproduction and bone growth and the maintenance of brain function [11,12]. However, in concentrations above the limit permitted by law (CONAMA), Mn can cause negative effects on human health.

Excessive amounts of Al and Mn pose long-term risks for human health. Al and Mn induce changes in the morphology of organs as well as cause cytotoxicity and genotoxicity [13,14,15,16,17]. Moreover, these metals may accumulate in the central nervous system and increase the risk of developing diseases, such as Alzheimer’s disease, Parkinson’s disease, and manganism syndrome [12,18,19,20].

Since the Chinese hamster ovary (CHO) cell line is representative or predictive of the human micronucleus (MCN) response, in the present study, CHO and CHO-XRS5 were used to test the hypothesis that Al and Mn induce cytotoxicity, genotoxicity, and mutagenicity. CHO cells, derived from the normal ovarian epithelial cells of Chinese hamsters, are well-characterized and have a short generation time (12–14 h) and a small number of chromosomes (2*n* = 20–22) compared to the human karyotype (2*n* = 46) [21]. CHO cells are highly recommended as a model for the in vitro assessment of toxicity and genotoxic potential as the results obtained with it mimic those of human cells [22]. Furthermore, the Organization for Economic Cooperation and Development (OECD) guidelines [23] favor the use of cell lines from Chinese hamsters. CHO-XRS cells are deficient in their ability to repair double-stranded DNA and are more efficient in the repair of single-strand breaks [24,25]. This inability prevents proper recombination and leads to changes in the DNA, which makes it a valuable tool for the evaluation of the mechanisms related to DNA break repair and chromosomal abnormalities caused by the toxic action of Al and Mn [26,27].

In our previous study, we assessed the quality of groundwater in two cities in Mato Grosso do Sul, Brazil [28] since its population is supplied entirely by groundwater, which is normally consumed without prior treatment. We identified Al and Mn concentrations above the maximum limits allowed (0.2 and 0.1 mg/L, respectively) by regulation 396/2008 of the National Council for the Environment [29], which specifies the maximum limits allowed for metals in groundwater intended for human consumption. To identify the potential effects caused by Al and Mn in the populations of these cities, the objective of this study was to use the same concentrations of Al and Mn detected previously and assess their cytotoxic and genotoxic effects in hamster ovary cell lines and their mutagenic effects through the *Salmonella* microsome assay.

## 2. Materials and Methods

### 2.1. Cell Culture

#### 2.1.1. Cell Lines and Treatments

Hamster ovary cell lines CHO and CHO-XRS5 were cultured in HAM-F10 (Sigma-Aldrich, St. Louis, MO, USA) and Dulbecco’s Modified Eagle’s Medium (DMEM) powder (high glucose) (Gibco, Carlsbad, CA, USA) supplemented with 10% fetal bovine serum (FBS) (Gibco, Carlsbad, CA, USA), 5 mg of penicillin, 5 mg of streptomycin, 10 mg of neomycin (Gibco, Carlsbad, CA, USA), and 3.7 g/L sodium bicarbonate. The cells were incubated at 37 °C under 5% CO_2_.

The concentrations of Al (0.2, 0.4, 0.6, 0.8, 1.0, and 2.0 mg/L) and Mn (0.1, 0.15, 0.3, 1.0, 1.5, and 3.0 mg/L) used in this study were based on the results of our previous study [28], in which we verified levels of Al and Mn within this concentration range in groundwater intended for human consumption. Stock solutions (1000 mg/L) of Al and Mn (SpecSolô, SEM-682, Jacareí, Brazil) were used. Mitomycin C (2 µg/L) (Sigma-Aldrich, St. Louis, MO, USA) was used as a positive control (PC) to the cytokinesis-block micronucleus cytome (CBMN Cyt) and comet assay, and culture medium alone was used as the negative control (NC).

#### 2.1.2. Cytotoxicity Assay

Cell viability was assessed through a colorimetric assay using 3-(4,5-dimethylthiazol-2-yl)-2,5-diphenyltetrazolium bromide (MTT, Sigma-Aldrich, St. Louis, MO, USA). For each concentration of the metals evaluated, three independent experiments were carried out in triplicates. For this, CHO and CHO-XRS5 cells (6 × 10^3^ cells/mL) were treated with Al and Mn for 24 and 72 h at 37 °C with 5% CO_2_. To ensure the same cell density until the end of the experiment, these metals were diluted in HAM-F10 + DMEM without FBS. After the treatment periods, MTT (0.5 mg/mL) was added to each well. The resulting supernatant was discarded, and dimethyl sulfoxide (DMSO, VETEC, Rio de Janeiro, Brazil) was added to solubilize the formazan crystals formed. Cell viability was calculated using the following formula:

Cell viability (%) = (Abs_treated_ cells/Abs_control_) × 100 (1)

#### 2.1.3. CBMN Cyt Assay

The MCN test with cytokinesis-block was performed according to the method described by Oliveira et al. [30] with some modifications. CHO and CHO-XRS5 cells (4 × 10^5^ cells/mL) were treated with Al (0.2–2.0 mg/L) and Mn (0.1–3.0 mg/L) in HAM-F10 + DMEM supplemented with FBS for 24 and 72 h at 37 °C with 5% CO_2_. After the treatments, the cells were incubated with Cytochalasin B (6 µg/mL; (Sigma-Aldrich, St. Louis, MO, USA) for 20 h. Subsequently, the cells were treated with 1% sodium citrate, fixed in a methanol:acetic acid (3:1) solution, and then stained with 5% Giemsa (VETEC, Rio de Janeiro, Brazil). One thousand binucleated cells were analyzed for each condition. Two independent experiments with one biological replicate for each sample were performed, and two technical replicates (in slides) were analyzed for each biological replicate. Thus, 4000 binucleated cells (2000 cells from each replicate) in each tested concentration were scored to determine the number of MCNs, nucleoplasmic bridges, and nuclear buds, as described by Fenech [31].

#### 2.1.4. Alkaline Single-Cell Gel Electrophoresis Assay (Comet Assay)

The comet assay was performed according to the methods of Singh et al. [32], with some modifications. CHO and CHO-XRS5 cells (1 × 10^5^/mL) were treated with Al (0.2–2.0 mg/L) and Mn (0.1–3.0 mg/L) in HAM-F10 + DMEM supplemented with FBS for 24 and 72 h at 37 °C with 5% CO_2_. An aliquot (100 μL) of the cell suspension was added to low melting point agarose (0.5%) (Thermo Fisher Scientific, Vilnius, Lithuania) at 37 °C and placed on gelatin-coated slides with 1.5% agarose (Kasvi, São José dos Pinhais, Brazil). Afterward, the slides were immersed in a lysis solution (2.5 M NaCl, 100 mM Na_2_ EDTA, and 10 mM Tris with 10% DMSO and 1% Triton X-100) at 4 °C for 90 min. Next, the slides were transferred to a horizontal electrophoresis unit dipped in alkaline electrophoresis buffer (0.3 mol/L NaOH and 0.001 mol/L EDTA, pH > 13) for 20 min and were electrophoresed (37 V/cm, 300 mA) for 25 min. Subsequently, the slides were neutralized with 0.4 M Tris for 15 min, fixed in ethanol, and stained with ethidium bromide (0.02 mol/L).

The nucleoids were analyzed using the Lucia Comet Assay Single Stain software (Laboratory Imaging, Prague, Czech Republic). In each of the two independent experiments, 100 comets were measured (50 comets/slide from two replicate slides). Thus, 200 comets in each tested concentration were scored. The genotoxicity parameters used to quantify DNA damage were DNA percentage in *Tail* and *Tail Length*.

### 2.2. Ames Test

The mutagenic potential of Al (0.025, 0.05, 0.1, 0.2, 0.4, 0.6, 0.8, and 1.0 mg/plate) and Mn (0.0125, 0.025, 0.05, 0.1, 0.3, 1.0, and 1.5 mg/plate) samples was assessed using the pre-incubation method according to the methods of Mortelmans and Zeiger [33] and the OECD guideline 471 [34]. The assay was conducted in both the presence and absence of exogenous metabolism using the liver of Sprague Dawley rats. The experiment was carried out in triplicates using the *Salmonella enterica* serovar Typhimurium strains TA98 and TA100, standardized to obtain a cell suspension of 10^8^ cells/mL. In the tests without metabolic activation, the following PCs (Positive Controls) were used: 4-nitro-phenylenediamine (10 μg/plate) for the TA98 strain and sodium azide (2.5 μg/plate) for the TA100 strain. In the tests with metabolic activation, 2-aminoanthracene (0.625 μg/plate) was used for both strains. For the Negative Control, DMSO 5% (5 μL/plate) was used.

### 2.3. Statistical Analysis

For the cell culture tests, statistical analyses were performed using the RStudio platform [35]. The normality of all data was tested using the Shapiro–Wilk test. The results of the MTT and MCN tests were evaluated using the one-way analysis of variance parametric test and Tukey’s test. The Kruskal–Wallis non-parametric test and Dunn’s test were used for analyzing the comet assay results. The differences between the treatments and the NC were considered significant at *p* < 0.05 for all tests.

For the Ames test, the results were analyzed by using the Salanal statistical program (U.S. Environmental Protection Agency, Monitoring Systems Laboratory, Las Vegas, NV, USA, version 1.0, from the Research Triangle Institute, Raleigh, Durham e Chapel Hill, NC, USA) and adopting the model of Bernstein et al. [36]. The mutagenicity ratio (MR) was calculated according to the following formula:

(2) $$\mathrm{MR}=\frac{\mathrm{Number}\;\mathrm{of}\;\mathrm{induced}\;\mathrm{revertants}}{\mathrm{Number}\;\mathrm{of}\;\mathrm{spontaneous}\;\mathrm{revertants}}$$

Considering that the tests were performed in a single dose, the results were presented as qualitative data. The metals were considered potentially mutagenic when the MR was ≥ 2.0, negative when the MR was <2.0, and toxic when the MR was <0.7.

## 3. Results

### 3.1. Cytotoxicity

**Figure 1:**
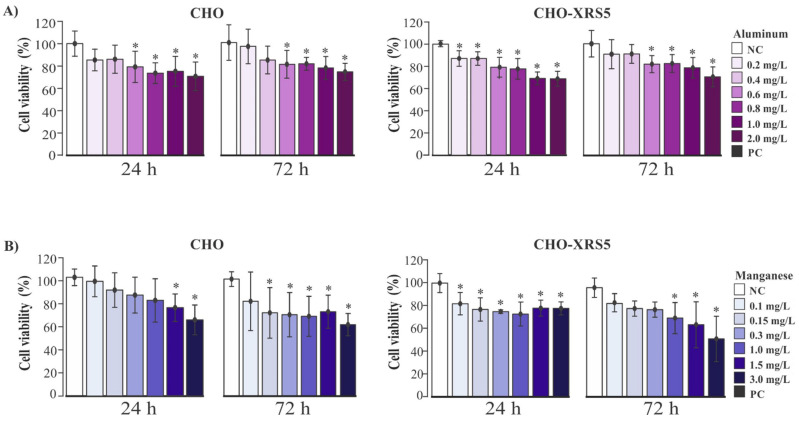
Viability (%) of Chinese hamster ovary (CHO) and CHO-XRS5 cells. Cells were treated with different concentrations of aluminum (**A**) and manganese (**B**) for 24 and 72 h. Data are expressed as means ± standard deviation of three independent experiments performed in triplicates. * *p* < 0.05 compared to the negative control by the analysis of variance test followed by Tukey’s test. NC, negative control.

The viability of CHO and CHO-XRS5 cells was reduced upon exposure to Al and Mn for 24 and 72 h, indicating their cytotoxic effects (Figure 1A,B).

The viability of CHO cells reduced significantly with Al concentrations ≥0.6 mg/L when compared to the NC during the 24 and 72 h periods of assessment (Figure 1A). The viability of CHO-XRS5 cells reduced with Al concentrations ≥0.2 mg/L and ≥ 0.6 mg/L for the 24 and 72 h time periods, respectively (Figure 1A).

The viability of CHO cells was reduced significantly at Mn concentrations > 1.5 mg/L when compared to the NC for the 24 h treatment; however, the viability of these cells was affected from a concentration of 0.15 mg/L for the 72 h treatment, i.e., greater toxicity was detected upon exposure for a longer duration (Figure 1B). The viability observed in CHO-XRS5 cells upon treatment with Mn was reduced at the lowest concentration (0.1 mg/L) after 24 h of treatment (Figure 1B). All concentrations of Al and Mn used for the cell viability test were used for analysis of the genotoxic effects of Al and Mn because none of them reduced the cell viability by more than 50%.

### 3.2. MCN Test

**Figure 2:**
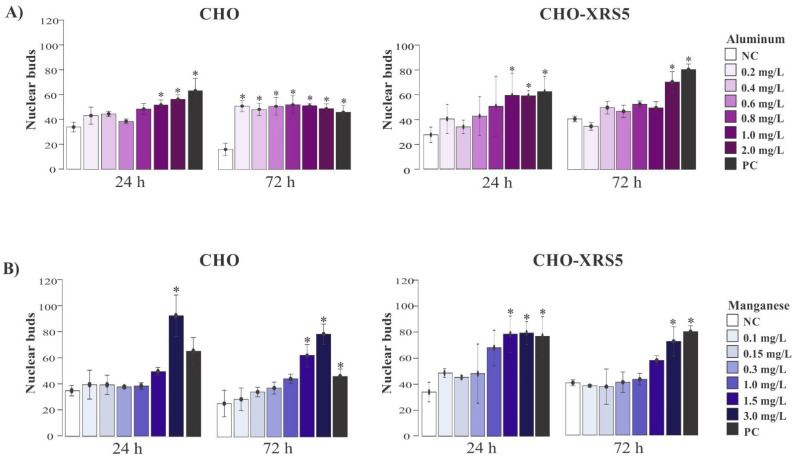
Effect of aluminum (**A**) and manganese (**B**) on nuclear bud formation in binucleated Chinese hamster ovary (CHO) and CHO-XRS5 cells after 24 and 72 h of incubation. Data are expressed as means ± standard deviation of two independent experiments. * *p* < 0.05 compared to the negative control via analysis of variance followed by Tukey’s test. NC, negative control; PC, positive control.

In CHO cells, the number of nuclear buds increased significantly at Al concentrations above 1.0 mg/L after 24 h of treatment compared to that of the NC. After 72 h of treatment, the lowest Al concentration (0.2 mg/L) tested was able to induce nuclear bud formation in these cells (Figure 2A).

In CHO-XRS5 cells, the number of nuclear buds increased significantly at Al concentrations above 1.0 mg/L after 24 h of treatment compared to that in the NC and in the highest Al concentration (2.0 mg/L) after 72 h of treatment (Figure 2A). In CHO cells, the number of nuclear buds was significantly different in the cells treated with 3.0 and 1.5 mg/L Mn for 24 and 72 h, respectively, compared to that in the NC (Figure 2B), whereas in CHO-XRS5 cells, this difference was observed from 1.5 and 3.0 mg/L after 24 and 72 h, respectively (Figure 2B). Al and Mn did not cause any significant increase in the number of nucleoplasmic bridges in either cell type.

**Figure 3:**
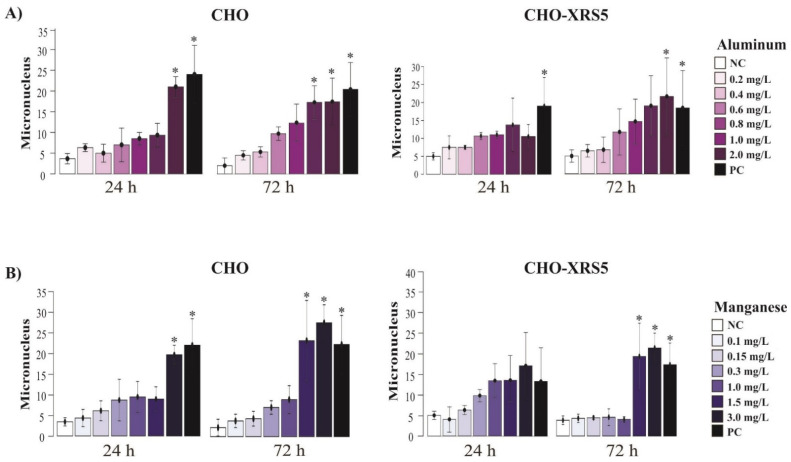
Effect of aluminum (**A**) and manganese (**B**) on micronucleus formation in binucleated Chinese hamster ovary (CHO) and CHO-XRS5 cells after 24 and 72 h of incubation. Data are expressed as means ± standard deviation of two independent experiments. * *p* < 0.05 compared to the negative control via analysis of variance followed by Tukey’s test. NC, negative control; PC, positive control.

In the present study, there was an increase in the MCN in CHO cells treated with Al compared to that in the NC during the two treatment periods. The increase in the MCN was significantly different compared to that in the NC in the 2.0 mg/L sample during the 24 h treatment period and in the 1.0 and 2.0 mg/mL samples in the 72 h treatment period (Figure 3A).

CHO-XRS5 cells exposed to Al exhibited a significant increase in the number of MCNs only at the highest test concentration of the metal (2.0 mg/L) during the 72 h treatment (Figure 3A). There was a significant increase in the number of cells with MCNs for CHO cells treated with Mn only at 3.0 mg/L after 24 h, whereas an increase was observed at concentrations ≥ 1.5 mg/L after 72 h of treatment (Figure 3B). In CHO-XRS5 cells, there was no significant difference in the number of MCNs during the 24 h treatment period; however, after 72 h of treatment, there were significant differences at concentrations ≥ 1.5 mg/L (Figure 3B).

### 3.3. Comet Assay

**Figure 4:**
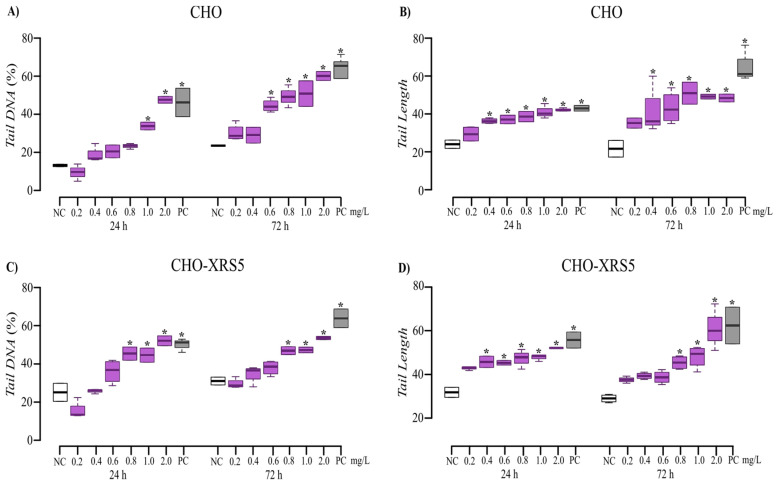
*Tail DNA* (**A**,**C**) and *Tail Length* (**B**,**D**) measured in CHO (**A**,**B**) and CHO-XRS5 (**C**,**D**) exposed to aluminum (violet) for 24 and 72 h. The values are expressed as medians and interquartile deviations of two independent experiments. * *p* < 0.05 compared to the NC by the Kruskal–Wallis test followed by Dunn’s test. NC, negative control (white); PC, positive control (mitomycin C; gray).

Al induced a significant increase (*p* < 0.05) in *Tail DNA* proportion in CHO cells with increasing concentration and exposure time (Figure 4A). Regarding *Tail Length*, there was a significant difference between the treatments and the NC at concentrations ≥0.6 mg/L during both periods of exposure (Figure 4B).

**Figure 5:**
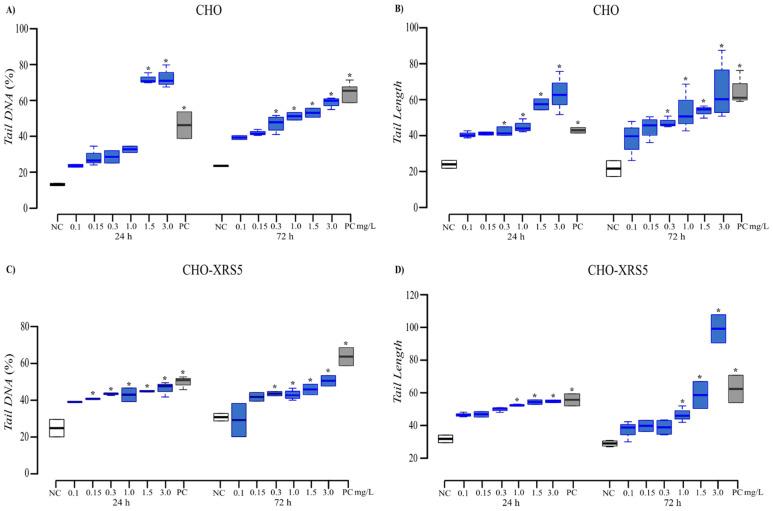
*Tail DNA* (**A**,**C**) and *Tail Length* (**B**,**D**) measured in CHO (**A**,**B**) and CHO-XRS5 (**C**,**D**) cells exposed to manganese (blue) for 24 and 72 h. The values are expressed as medians and interquartile deviations of two independent experiments. * *p* < 0.05 compared to the negative control by the Kruskal–Wallis test followed by Dunn’s test. NC, negative control (white); PC, positive control (mitomycin C; gray).

In CHO-XRS5 cells, there was a significant difference between the treated cells and the NC for *Tail DNA* percentage at Al concentrations ≥0.8 mg/L during both test periods (Figure 4C) and for *Tail Length* at 0.4 and 0.8 mg/L for the 24 and 72 h treatments, respectively (Figure 4D). In CHO cells treated with Mn, there was a significant increase in *Tail DNA* percentage with increasing concentrations and exposure time (Figure 5A), while for *Tail Length* there was a significant difference at concentrations ≥0.3 mg/L during both assessment periods (Figure 5B).

In the CHO-XRS5 cells, regarding the *Tail DNA* percentage, the negative effects of Mn were significant at concentrations ≥ 0.15 mg/L for the 24 h treatment and at concentrations ≥ 0.3 mg/L for the 72 h treatment (Figure 5C). A difference in *Tail Length* was observed at concentrations ≥ 1.0 mg/L for both periods (Figure 5D).

### 3.4. Salmonella Microsome Assay (Ames Test)

In this study, we evaluated two bacterial strains (*S. Typhimurium* TA98 and TA100) with and without metabolic activation (S9) to test the mutagenicity of Al and Mn. The strain TA98 was used for the detection of frameshift mutations, while the strain TA100 was used to detect base pair substitutions, primarily at guanine:cytosine (G:C) pairs.

As shown in Table 1, no mutagenic effect (MR > 2) was observed in strains TA98 and TA100 at any concentration of Al, either in the presence or absence of S9. We observed an MR < 0.7 in the range tested (0.2 to 1 mg/L), demonstrating the cytotoxic effect of Al in the strains evaluated. Therefore, we reduced the concentration range of the evaluated metal to verify its mutagenic potential, and all concentrations tested (0.025, 0.05, and 0.01 mg/L) yielded negative results (MR < 2) in both strains with and without metabolic activation (Table 1 and Table S1). However, the concentrations 0.025, 0.05, and 0.01 mg/L of Al in the presence and absence of S9 to the TA 100 strain compared to NC showed a tendency to become mutagenic, as they significantly increased the number of reversing colonies.

At the higher metal concentrations, fewer bacterial colonies grew on the Petri dish. As observed by the Ames test, Al also showed a cytotoxic effect in the assay with cell lines CHO and CHO-XRS5, from the concentrations 0.6 and 0.2 mg/L, respectively (Figure 1). According to the results for Mn, no test concentration caused a positive mutagenic response when compared with the control in the two *S. Typhimurium* strains, with or without metabolic activation (Table 2). A cytotoxic effect was found at concentrations from 0.1 to 1.5 mg/L; however, when the concentration range was reduced (0.0125, 0.025, and 0.05 mg/L), we observed negative results (MR < 2) in both strains with and without metabolic activation (Table 2 and Table S1). The 0.0125 mg/L concentration with or without metabolic activation for the TA98 strain also significantly increased the number of reversing colonies in relation to the NC, demonstrating the tendency to become mutagenic. A similar result was observed for the strain TA 100 at the concentration of 0.05 mg/L in the assay with metabolic activation.

In general, Mn demonstrated a cytotoxic effect as the concentration increased (Table 2) and when compared to that in the cellular assay, Mn demonstrated cytotoxicity in CHO cells from a concentration of 0.15 mg/L for 72 h of treatment and in CHO-XRS5 cells from 0.1 mg/L for 24 h of treatment (Figure 1).

## 4. Discussion

Metals are non-biodegradable chemical contaminants that accumulate in living organisms that can be toxic to plants and microorganisms as well as affect human health [37]. Therefore, studies that evaluate their cytotoxicity, genotoxicity, and mutagenicity in organisms are extremely important. Thus, bioassays with plants [38,39,40], animals [16,41,42], and human cells [5,16,43,44,45] are important tools for the assessment of the responses of organisms when exposed to metals.

Both in vitro and in vivo studies have demonstrated that exposure of cells to Al leads to reactive oxygen species (ROS) formation, lipid peroxidation, and oxidative damage of mitochondrial proteins, which results in cytotoxicity [46,47,48]. Concentration-dependent cytotoxicity of Al at concentrations similar to those used in this study (0.2–1.0 mg/L) has previously been observed in the lymphocytes and thymocytes of rats, with a significant reduction in cell viability directly proportional to the increase in Al concentration [49].

The reduction in cell viability observed in cells exposed to Mn after 24 h has also been found in studies conducted using other cell lines, where higher cytotoxicity is observed after a 24 h treatment period [17,50,51]. The mechanisms associated with the loss of cell viability due to Mn exposure still need to be clarified; however, oxidative stress due to the generation of ROS upon induction by Mn is related to cell death in several cell lines [52,53,54,55,56]. To elucidate the cytotoxic and genotoxic effects of Al and Mn, a comparison was made between the wild type strain CHO and CHO-XRS5, a cell line lacking the ability to repair double-strand breaks efficiently. The reduction in cell viability upon exposure to the lowest concentration (0.2 and 0.1 mg/L) of Al and Mn was observed only in CHO-XRS5 cells after a 24 h treatment period and may be due to the inefficiency of these cells to repair double-strand DNA breaks [24,25].

In this study, the parameters of nuclear buds, nucleoplasmic bridges, and MCN were analyzed to assess the genotoxic damage that Al and Mn can cause in cells. In general, Al and Mn caused significant genotoxic damage at the highest concentrations tested (above 1.0 mg/L) with the exception of the formation of nuclear buds, in which case a significant increase was observed in CHO-XRS5 cells after 72 h of treatment with the lowest Al concentration tested (0.2 mg/L). Neither Al nor Mn caused a significant increase in the number of nucleoplasmic bridges in either of the cell lines tested; however, a study conducted by Porte Alcon et al. [52] reported an increase in the number of cells with nucleoplasmic bridges and micronuclei when murine microglial cells (BV 2) were exposed to high Mn concentrations (13.75 and 41.25 mg/L).

Al induces DNA damage and may lead to cytotoxic, genotoxic, or carcinogenic alterations in cells [57]. The genotoxic effect of AlCl_3_ has been reported in human lymphocytes at concentrations > 0.135 mg/L after 72 h of exposure, with higher numbers of MCNs being observed at concentrations of 0.27 and 0.54 mg/L [16]. In relation to Mn, a significant difference in the number of MCNs between treatments and the NC was also observed in murine microglial cells (BV-2) [52] at concentrations of 13.75 and 41.25 mg/L Mn, which are higher than the concentrations used in this study. The significant increase in the number of MCNs in the CHO and CHO-XRS5 cell lines occurred at the highest Mn concentrations. An increase in DNA damage, as evidenced by the presence of nuclear buds and MCNs, may be a consequence of oxidative stress and/or increased nuclear expression of cH2AX caused by exposure to Mn [52].

The comet assay showed that Al induced a significant increase in DNA damage to cells with increasing concentration and exposure time (Figure 4). Celik et al. [57] demonstrated a positive correlation between increased oxidative stress and DNA damage (as measured by the comet assay) in cells treated with Al. However, studies using more specific molecular techniques that assess epigenetic effects would be relevant for elucidating the mechanisms responsible for DNA damage due to Al exposure in CHO and CHO-XRS5 cells.

Treatment with Mn induced a significant increase in DNA damage to cells with increasing concentrations for both cell lines, as evidenced by different DNA damage parameters. DNA damage was also detected in human lymphocytes exposed to Mn in different phases of the mitotic cycle [58] and in human neuroblastoma cells (SH-SY5Y) exposed to Mn for 24 h [17]. In the present study, when comparing the genotoxic damage between the CHO and CHO-XRS5 cell lines, no greater amount of genotoxic damage was observed in the CHO-XRS5 cell line compared to that in the CHO cell line, which was not expected due to the inefficiency of these cell types in repairing double-stranded DNA breaks.

No mutagenic effect (MR > 2) was observed in strains TA98 and TA100 treated with Al and Mn at any test concentration. The range tested (0.2–2.0 and 0.1–1.5 mg/L) had an MR < 0.7, demonstrating the cytotoxic effect of the compound in the strains evaluated. Our results are consistent with those of previously published studies for both strains regarding treatment with Al. Saraç et al. [59] showed no mutagenic effects in the Ames test for the concentrations of AlCl_3_ evaluated (0.025, 0.25, and 1.25 mg/mL plate). In another study, Ahn and Jeffery [13] verified that Al did not cause mutagenic effects at concentrations of 0.3 and 3.0 mg/L but was suggested as toxic to the TA98 strain. This toxicity observed by Ahn and Jeffery [13] should be explained because when the viability of cells was evaluated, Al caused a decrease in viability compared to that in the NC. Based on the results of our study, we could infer that the cytotoxic effects on the strains evaluated interfered in the detection of mutagenic effects since Al and Mn could have caused DNA damage that the strains were unable to repair, which caused them to stop growing, thereby preventing the detection of mutagenic effects.

## 5. Conclusions

Al and Mn at concentrations detected previously in groundwater (the maximum limit permitted by law as well as higher values) and evaluated in this study were shown to have cytotoxic and genotoxic effects in hamster ovary cell lines. This study identified the cytotoxic effects of Al and Mn, as evidenced in the *Salmonella* microsome assay, even at concentrations that were within the legally permissible limits. These results may help hasten the review of established regulatory standards of groundwater for human consumption.

## Figures and Tables

**Table 1:** Mutagenicity ratio (MR) of the Al expressed by the mean of reversals/plate ± standard deviation to TA98 and TA100 strains of *S. Typhimurium* with (S+) and without (S-) metabolic activation.

Concentrations (mg/plate)	TA 98		TA 100	
	S+ (MR)	S− (MR)	S+ (MR)	S− (MR)
NC	20.33 ± 1.24	23.33 ± 1.24	122.33 ± 1.88	111.33 ± 1.69
0.025	19.66 ± 0.44 (0.96)	28.66 ± 3.55 (1.22)	149.70 ± 8.44 * (1.22)	149.33 ± 1.11 * (1.34)
0.05	18.66 ± 0.44 (0.93)	21.00 ± 3.33 (0.90)	132.70 ± 1.55 * (1.08)	134.66 ± 3.11 * (1.20)
0.1	24.00 ± 1.30 (1.2)	27.00 ± 2.00 (1.15)	163.00 ± 4.00 * (1.33)	136.50 ± 4.50 * (1.22)
0.2	10.00 ± 0.81 (0.37)	7.33 ± 0.47 (0.33)	13.00 ± 0.81 (0.18)	26.00 ± 4.00 (0.33)
0.4	-	10.33 ± 0.47 (0.46)	18.00 ± 1.41 (0.25)	26.50 ± 1.50 (0.34)
0.6	-	11.00 ± 1.41 (0.50)	17.00 ± 1.63 (0.23)	15.66 ± 0.94 (0.20)
0.8	-	9.33 ± 0.47 (0.42)	16.33 ± 1.24 (0.23)	17.33 ± 1.24 (0.22)
1.0	-	7.66 ± 0.94 (0.34)	17.00 ± 1.41 (0.23)	19.00 ± 1.63 (0.24)
PC	321.00 ± 11.00 *	1315.00 ± 8.00 *	1338.00 ± 11.00 *	1428.00 ± 6.00 *

Negative control (NC): dimethyl sulfoxide 5%; positive control (PC): 4-nitro-o-phenylenediamine (10 μg/plate); sodium azide (2.5 μg/plate); 2AA-aminoanthracene (2.5 µg/plate); -, no colony growth; * *p* < 0.05.

**Table 2:** Mutagenicity ratio (MR) of the manganese expressed by the mean of reversals/plate ± standard deviation to TA98 and TA100 strains of *S. Typhimurium* with (S+) and without (S-) metabolic activation.

Concentrations (mg/plate)	TA 98		TA 100	
	S+ (MR)	S− (MR)	S+ (MR)	S− (MR)
NC	20.33 ± 1.24	23.33 ± 1.24	122.33 ± 1.88	111.33 ± 1.69
0.0125	25.66 ± 0.88 * (1.26)	29.66 ± 0.88 * (1.27)	90.00 ± 6.66 (0.73)	87.66 ± 1.88 (0.78)
0.025	20.33 ± 0.44 (1.00)	22.66 ± 1.11 (0.97)	108.66 ± 2.22 (0.88)	92.33 ± 2.22 (0.82)
0.05	25.00 ± 1.33 (1.22)	26.66 ± 3.11 (1.14)	148.00 ± 5.33 * (1.20)	108.33 ± 2.22 (0.97)
0.1	10.66 ± 1.24 (0.39)	8.33 ± 0.94 (0.37)	18.00 ± 5.00 (0.25)	12.66 ± 0.47 (0.16)
0.3	14.00 ± 2.16 (0.51)	9.33 ± 0.47 (0.42)	-	-
1.0	8.66 ± 1.88 (0.32)	6.50 ± 0.50 (0.29)	-	-
1.5	3.33 ± 0.94 (0.12)	7.00 ± 0.81 (0.31)	-	-
PC	321.00 ± 11.00 *	1315.00 ± 8.00 *	1338.00 ± 11.00 *	1428.00 ± 6.00 *

Negative control (NC): dimethyl sulfoxide 5%; positive control (PC): 4-nitro-o-phenylenediamine (10 μg/plate); sodium azide (2.5 μg/plate); 2AA-aminoanthracene (2.5 µg/plate); -, no colony growth; * *p* < 0.05.

## Data Availability

All the data in the current study could be available by contacting the corresponding author.

## Supplementary Materials

The following are available online at https://www.mdpi.com/article/10.3390/toxics9070153/s1, Table S1: Mutagenicity (MR) results obtained in the Salmonella/microsome assay from the concentrations of Al and Mn using the *S. Typhimurium* strains TA98 and TA100 in the presence and absence of metabolic activation system (S9).
