# Supplementary material for: Acute Toxic and Genotoxic Effects of Aluminum and Manganese Using In Vitro Models
Source: Toxics. 2021 Jun 30;9(7):153. doi: 10.3390/toxics9070153 (PMC8309840; doi:10.3390/toxics9070153)
Supplement: Supplementary file 1 [file toxics-09-00153-s001.zip › toxics-1179913-supplementary.pdf]

Supplementary Materials: Acute Toxic and Genotoxic Effects of Aluminum and Manganese Using In Vitro Models

Luiza Flavia Veiga Francisco, Débora da Silva Baldivia, Bruno do Amaral Crispim, Syla Maria Farias Ferraz Klafke, Pamella Fukuda de Castilho, Lucilene Finoto Viana, Edson Lucas dos Santos, Kelly Mari Pires de Oliveira and Alexeia Barufatti

Table S1. Mutagenicity (MR) results obtained in the Salmonella/microsome assay from the concentrations of Al and Mn using the S. Typhimurium strains TA98 and TA100 in the presence and absence of metabolic activation system (S9).

Metals	Concentrations (mg/plate)	TA 98		TA 100	
		S+	S-	S+	S-
Aluminum	0.025				
	0.05				
	0.1				
	0.2				
	0.4				
	0.6				
	0.8				
	1.0				
Manganese	0.0125				
	0.025				
	0.05				
	0.1				
	0.3				
	1.0				
	1.5				

Legend:
Toxic (MR <0.7) Negative (MR <2)
